# Gibberellin delays metabolic shift during tomato ripening by inducing auxin signaling

**DOI:** 10.3389/fpls.2022.1045761

**Published:** 2022-11-14

**Authors:** Me-Hea Park, Siva Kumar Malka

**Affiliations:** Postharvest Research Division, National Institute of Horticultural and Herbal Science, Wanju-gun, South Korea

**Keywords:** auxin signaling, ethylene, gibberellin, hormone interaction, metabolic shift, ripening, sucrose, tomato

## Abstract

Fruit ripening involves the dynamic interaction of phytohormones. Ethylene (ET) and gibberellin (GA) antagonistically affect fruit ripening. However, the mechanism of GA and its potential interaction with ET during fruit ripening remain unknown. To identify the potential molecular mechanism of ET and GA interplay in tomato (*Solanum lycopersicum* L.) fruit ripening, transcriptome and metabolomic profiling was carried out in tomato fruit treated with GA, ET or the combination of the two hormones (GA+ET). ET accelerated fruit ripening with the simultaneous repression of auxin signaling. In contrast, gibberellin delayed ripening by the upregulation of auxin signaling. ET signaling and response was inhibited by GA or combined with ET. At the metabolite level, while GA treatment inhibited metabolite shift during ripening, ET treatment promoted. In the combined hormone treatment, ET reduced or recovered GA inhibitory effect on specific metabolites. This study provided insight into ET and GA interaction, highlighting the importance of auxin signaling in metabolic shifts during tomato ripening progression.

## Introduction

Fruit ripening involves dynamic interactions between phytohormones. Ethylene (ET) promotes ripening, and its role in this process is well established. In contrast, gibberellin (GA) slows ripening; manipulation of endogenous GA levels affects ripening progression (García-Hurtado et al., 2012; Li et al., 2019). However, the mechanism of GA and its possible interaction with ET during fruit ripening remains unknown. Understanding hormonal interactions in ripening is essential to regulating the ripening process and the transition to fruit spoilage, the target of a multi‐billion‐dollar industry.

During the onset of ripening, there is a significant shift in the relative hormone levels in the fruit, with a decrease in auxin, GA, and cytokinin levels and an increase in abscisic acid and ET levels (Kumar et al., 2014). In climacteric fruits that continue to ripen after harvest, ET is the major cue that controls several aspects of ripening. Altering ET at the level of its biosynthesis, perception, signal transduction, or gene transcription negatively affects fruit ripening (Kumar et al., 2014). Two ET production systems operate during fruit development and ripening. In the immature stages, ET biosynthesis is mediated by system 1 (autoinhibitory), whereas system 2 (autocatalytic) takes over during ripening. ET production in these two systems is controlled *via* the differential regulation of *ACC synthase (ACS)* and *ACC oxidase (ACO)* genes (Barry et al., 2000). Furthermore, ET application can accelerate the ripening process (Alexander & Grierson, 2002). GA is a growth-promoting hormone; the regulatory role of GA in fruit development has been extensively studied (Yamaguchi, 2008). GA content decline during fruit maturation to ripening; however, its exogenous application can delay fruit ripening (Kumar et al., 2014). Moreover, overexpression of *SlGA2ox1*, a GA catabolism gene, induced ripening, whereas transgenic tomato overexpressing GA biosynthetic gene *GA20ox* delayed fruit ripening time (García-Hurtado et al., 2012; Li et al., 2019). Previous studies showed that GA and auxin mediate the duration of fruit development versus ripening through crosstalk with abscisic acid, the primary ripening hormone in non-climacteric fruits (Liao et al., 2018). The study suggests that GA may interact with ET during the transition from the early growth phase to ripening. However, our knowledge of the molecular response of GA alone or in combination with ET is limited in climacteric fruits.

In this study, tomatoes were treated with ET, GA, or a combination of both (GA+ET) at the mature green to breaker stage. The hormonal effect was characterized at physiological, transcriptome, and metabolomic levels.

## Materials and methods

### Plant materials and treatments

Cherry tomato (*Solanum lycopersicum* L. “Betatini”) fruits at the mature-green to breaker stages were harvested during summer in Jungyeum, South Korea. Disease-free and intact fruits were sterilized with 2% sodium hypochlorite solution and washed with tap water twice. After air drying at room temperature and removing pedicels, the fruits were divided into four groups and treated with ET, GA, or a combination of both. For ET treatment, the fruits were dipped in 1 mM ethephon (Inbio Corp, Jecheon, South Korea) solution for 5 min under vacuum at 30 kPa. For GA treatment, the fruits were dipped in 0.5 mM GA_3_ solution [prepared in ethanol/distilled water (1:1000, v/v) containing 0.1% (v/v) Tween-20; Sigma-Aldrich, St Louis, MO, USA] for 15 min. The fruits were sequentially treated with GA, air dried, and then treated with ET for the combined treatment. The fruits treated with sterile water served as control samples. Following treatment, the fruits were kept in darkness at 20 ± 2°C with 90% ± 5% relativity humidity (RH) for 10 days.

### Measurement of plant hormones

ET production was analyzed using a gas chromatograph (Agilent 7890b, Billerica, MA, USA). One milliliter of gas was sampled using a syringe from a 2 L container containing four fruits from each treatment and sealed for 2 h. The injection and column temperatures were set to 110°C and 70°C, respectively. The thermal conductivity detector and flame ionization detector used for the CO_2_ and ET measurements were set at 150°C and 250°C, respectively.

GA_3_ content was measured following the method described by (Ryu et al., 2020) with minor modifications. GAs were extracted from 100 mg freeze-dried powder of pericarp tissues and suspended in 100% methanol containing 400 ng of 2H-labelled GA4 (d2-GA4) as an internal standard at 4°C for at least 12 h. The samples were centrifuged and the supernatant was filtered through a nylon syringe filter with a pore size of 0.45 μm (Sartorius, SeongNam-Si, South Korea) and then drawn through a Sep-Pak C18 cartridge (Waters, Milford, MA, USA) equilibrated with 100% methanol. The extracts were dried using a rotary evaporator at 40°C and then redissolved in 200 μL of 100% methanol for further analysis. GAs were identified in a Liquid chromatography-mass spectrometry (Thermoelectron, San Jose, CA, USA) using a high-performance liquid chromatography (HPLC) system (Shiseido). Liquid chromatography separation was performed on a Unison UK-C18 column (2.0 × 50 mm, 3 μm; Imtakt, Portland, OR, USA). GA_3_ was quantified based on the peak area ratios of the analyst to the corresponding internal standard.

### Fruit quality evaluation

Fifteen fruits per treatment were sampled to assess fruit quality. Skin color was monitored using a color difference meter (Minolta CR-400, Japan) and reported based on Hunter’s scale: redness (a*). Firmness was analyzed using a texture analyzer (TA Plus Lloyd Instruments Ltd., UK) at the speed of 2 mm/s with a 5-mm diameter plunger head.

### Carotenoid analysis

Lycopene content was extracted and subsequently analyzed on the AQUITY UPLC H-Class system (Agilent Technologies Inc., Santa Clara, CA, USA) equipped with a HALO 160 Å C30 (2.1 × 50 mm, pre-column 2.1 × 5 mm; Wilmington, DE, USA). Dried powder (50 mg) of pericarp tissues was extracted with acetonitrile: MeOH (4:1). The HPLC conditions were as follows: column temperature, 31°C; detection wavelength, 450 nm; flow rate, 0.7 mL/min; and injection volume, 2 μL. Carotenoids were analyzed *via* gradient elution (70 → 100%) of the mobile phase solvents A (acetonitrile:methanol (75:25, v/v)) and B (methanol). Compounds were identified by comparing their elution times with those of verified standards.

### Transcriptome analysis

RNA from pericarp tissues pooled from five fruits was isolated using the cetyltrimethylammonium bromide protocol for each treatment. Library preparation and RNA sequencing (RNA-Seq) were performed by C & K Genomics (South Korea). Processed reads were aligned to the reference genome (*Solanum lycopersicum* version ITAG3.2) using HISAT software (ver 2-2.1.0 (Kim et al., 2015). Differentially expressed genes (DEGs) were identified using edgeR Bioconductor package based on the generalized linear model (GLM). False discovery rate < 0.05 significance cutoff was used for DEGs. Gene enrichment, functional annotation, and pathway analyses were performed using the DAVID 6.8 tool (Huang et al., 2007), and the Kyoto Encyclopedia of Genes and Genomes (KEGG) Database Hierarchical clustering analysis was performed using complete linkage and Euclidean distance as a measure of similarity to display expression patterns of DEGs with FC ≥1.

### Quantitative real-time PCR

Quantitative real-time PCR (qRT-PCR) was performed using a CFX96 TouchTM Real-Time PCR detection system (Bio-Rad, Hercules, CA, USA) as previously described by (Park et al., 2018). The transcripts were amplified using the iQTM SYBR Green Supermix (Bio-Rad) with specific primers (**Table S1**). qRT-PCR was performed under the following conditions: 95°C for 30 s, followed by 40 cycles of 95°C for 10 s and 55°C or 58°C for 40 s. Relative gene expression was calculated using the ΔΔCt method and normalized using the expression levels of the housekeeping gene *actin*. qRT-PCR analysis was carried out using at least three biological replicates and two technical replicates.

### Metabolite profiling using gas chromatography-mass spectrometry

Metabolite analysis was performed using GC–MS as previously described by (Lisec et al., 2006) with modifications. First, freeze-dried powder (50 mg) from pericarp tissues was vortexed with 1 mL of 80% methanol; then, the resulting mixture was sonicated for 30 min at 65°C and centrifuged for 10 min at 15,000 × g. Next, the supernatant (700 μL) was mixed with 20 μL of fluoranthene (5 g/L in water) was used as an internal standard. Subsequently, 150 µL of the extract solution was dried using a SpeedVac (Thermo Fisher Scientific, Waltham, MA, USA). After drying, the samples were incubated for 90 min at 30°C with 50 μL of methoxyamine hydrochloride (20.0 g/L in pyridine). Next, the samples were incubated for 30 min at 60°C with 50 L of N, O-bis(trimethylsilyl)trifluoroacetamide in 1% trimethylchlorosilane. Subsequently, an autosampler injected 1 μL of the sample was injected into the GC–MS ISQ LT system (Thermo Fisher Scientific, USA). A DB-5MS column (0.25 × 60 mm i.d., Agilent Technologies, USA) was used, and the oven temperature was set to increase from 50°C to 325°C at a rate of 5°C/min. The injector was in the split-less mode at 300°C. Helium was used as the carrier gas at a flow rate of 1.5 mL/min. The range of mass scans was from 35 to 650 m/z. The metabolite data were normalized and scaled and used for dendrogram construction, heatmap cluster analysis, and partial least squares-discriminant analysis (PLSDA) using MetaboAnalyst 3.0 software (www.metaboanalyst.ca).

### Statistical analyses

Values are presented as the mean ± standard error. Samples were subjected to analysis of variance, and significant differences were determined using Duncan’s multiple range test. All analyses were conducted using SAS v.9.2 (SAS Institute, Cary, NC, USA).

## Results

### Effects of ET and GA treatment on tomato ripening

**Figure 1:**
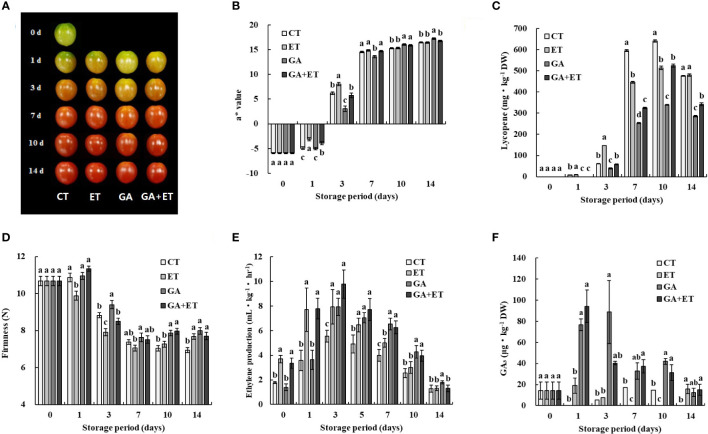
Effect of hormone treatments on tomato ripening. Changes in **(A)** color, **(B)** a* values, **(C)** lycopene content, **(D)** firmness, **(E)** ethylene production, and **(F)** GA_3_ content. Error bars represent standard error, and different letters on the graphs represent significant differences between the control and hormone treatments (Student’s *t*-test, P < 0.05). CT, control; ET, ethylene; GA, gibberellin; GA+ET, the combined hormone treatment.

Tomatoes at the mature green to breaker stage were treated with ET, GA, or both (GA+ET), and the ripening process was characterized by measuring the color, firmness, and ET production after the hormone treatments during 14 d of storage at room temperature. Hormone-induced color changes were visible on 3 d (**Figure 1A**). ET treatment accelerated fruit reddening and induced loss of firmness with consistently higher a* (redness, Hunter scale) values and lycopene content than those of the control (**Figures 1A–D**). The GA-treated fruits were firmer with delayed color transition, as evidenced by lower a* values and lycopene content than those of the control, ET-, and GA+ET-treated fruits (**Figures 1A–D**). Color development in the GA+ET-treated fruits was delayed compared with that in the control and ET-treated fruits but earlier than that in the GA-treated fruits (**Figures 1A–D**). Consistently, the firmness, a* values, and lycopene content in the GA+ET-treated fruits were largely similar to those in the control or in between to those in GA- or ET-treated fruits (**Figures 1A–D**). β-carotene levels did not show any variation after the hormone treatments, except on 14 d (**Figure S1**). A climacteric increase in ET was observed on 1 d in the ET- and GA+ET-treated fruits, but it was delayed by 2 d in the control and GA-treated fruits (**Figure 1E**). Similarly, endogenous GA levels peaked on day 1 in the GA-and GA+ET-treated fruits and steadily decreased thereafter (**Figure 1F**). In the control and ET-treated fruits, GA levels were largely similar but significantly lower than those in the GA and GA+ET-treated fruits (**Figure 1F**).

### Hormone-induced changes in transcriptomic profiles

**Figure 2:**
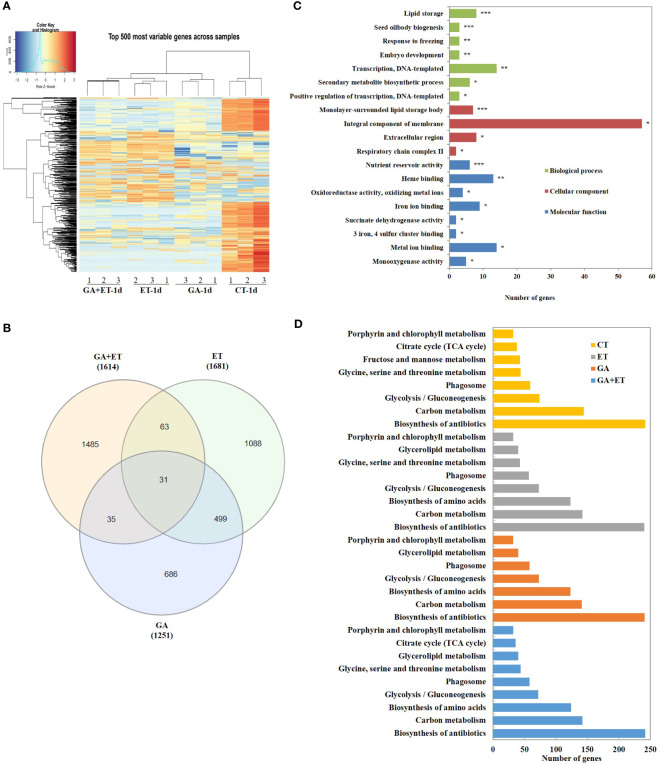
Transcriptome analysis of tomatoes with hormone treatment. Heatmap of differentially expressed genes (DEGs) in all samples **(A)**; Venn diagram of DEGs **(B)**; Functional categorization of DEGs **(C)**; KEGG pathway enrichment analysis of DEGs **(D)**. Analysis were performed using DAVID v.6.8. * represents *P* < 0.1, ** represents *P* < 0.01, *** represents *P* < 0.001. CT, Control; ET, ethylene; GA, gibberellin; the combined hormone treatment, GA+ET.

RNA sequencing was performed using pericarp tissues 1 d after hormone treatment. The heat map revealed dramatic changes after the hormone treatment (**Figure 2A**). In total, 4,546 DEGs were identified, of which 1,088, 686, and 1,485 DEGs were explicitly responsive to ET, GA, and GA+ET treatments, respectively (**Figure 2B**). The Gene Ontology terms annotated for the DEGs belonged to different functional groups, including cellular components, biological processes, and molecular functions (**Figure 2C**). KEGG enrichment analysis was performed to identify the distribution of DEGs among metabolic pathways. The top five enriched pathways were biosynthesis of antibiotics, carbon metabolism, biosynthesis of amino acids, glycolysis, and phagosomes (**Figure 2D**). All the upregulated and downregulated genes in response to the hormone treatments were provided in **Tables S2–S4**.

Concerning the early ET peak in ET-treated tomatoes, DEGs related to ET biosynthesis (*ACOs*) and signaling (ethylene response factors, *ERFs*) were upregulated in these fruits (**Table 1**). ET biosynthesis genes *ACS2* and *ACO6* were also induced in GA- and GA+ET-treated fruits, respectively; however, DEGs related to ET signaling and response were differentially regulated. For instance, *ERF4* and *ERF.C2* were suppressed, whereas *ERF.C3* and *ERF13* were induced in GA-treated fruits. In contrast, GA+ET treatment induced the expression of *ERF.B8*, *ERF.B10*, and ethylene-regulated nuclear-localized protein (*ERN*) but suppressed ET signaling components *ethylene insensitive 3 (EIN3)* and *EIN 3-like (EIL)*. In ET-treated fruits, DEGs encoding proteins associated with GA biosynthesis *(CYP88A)* and homeostasis *(CYP72A15)* were induced, indicating tight regulation of GA levels in these fruits. Exogenous GA might have reduced GA biosynthesis, as evidenced by the downregulation of the GA biosynthetic gene *GA20ox*. Additionally, GA alone or combined with ET suppressed GA catabolic genes *CYP72A15* and *GA2ox6*, respectively.

**Table 1:** Ethylene- and gibberellin-related differentially expressed genes (DEGs) expressed in hormone-treated tomatoes (ethylene, gibberellin, or a combination of both).

Treatment	Gene ID	Annotation	Fold change (log 2 ratio)
**Ethylene**	**Ethylene-related DEGs**		
	Solyc02g081190.3.1	*ACO4*	-1.04
	Solyc10g050970.1.1	*ERF.D4*	1.02
	Solyc04g009860.3.1	*ACO*	1.03
	Solyc12g005940.2.1	*AC02*	1.27
	Solyc03g093610.1.1	*ERF.A2*	1.28
	Solyc11g012980.1.1	*ERF.D9*	1.39
	Solyc06g054630.2.1	*ERF*	1.45
	Solyc07g026650.3.1	*ACO5*	1.77
	Solyc02g077370.1.1	*ERF.C5*	1.87
	Solyc08g078180.1.1	*ERF.A1*	2.37
	Solyc11g042560.1.1	*ERF021*	2.42
	Solyc06g068830.2.1	*ERF*	3.41
	**Gibberellin-related DEGs**
	Solyc07g062500.3.1	*CYP72A15*	1.16
	Solyc02g083880.3.1	*Gibberellin-regulated family protein*	1.17
	Solyc12g006460.2.1	*CYP88A*	1.21
**Gibberellin**	**Ethylene-related DEGs**		
	Solyc08g079750.3.1	*ACC synthase 10*	-1.52
	Solyc04g014530.1.1	*ERFC2*	-1.12
	Solyc03g006320.1.1	*ERF4*	-1.03
	Solyc09g066360.1.1	*ERF.C3*	1.04
	Solyc01g095080.3.1	*ACS2*	1.1
	Solyc01g090310.2.1	*ERF13*	3.85
	**Gibberellin-related DEGs**
	Solyc06g035530.3.1	*GA20ox-2*	-4.11
	Solyc12g099900.1.1	*Scarecrow-like 3*	-1.68
	Solyc07g062500.3.1	*CYP72A15*	-1.04
	Solyc02g089350.3.1	*Gibberellin-regulated family protein*	1.32
	Solyc07g056670.3.1	*Gibberellin 2-oxidase 1*	1.98
**Gibberellin+Ethylene**			
	**Ethylene-related DEGs**
	Solyc00g095860.1.1	*ACC synthase 1*	-7.56
	Solyc00g154980.1.1	*Ethylene insensitive 3 family protein*	-7.56
	Solyc01g009170.3.1	*Ethylene insensitive 3 family protein*	-3.49
	Solyc01g006650.2.1	*ETHYLENE-INSENSITIVE3-like 3*	-1.13
	Solyc02g036350.3.1	*ACO6*	1.58
	Solyc01g090370.2.1	*ERF.B10*	2.12
	Solyc01g090320.3.1	*ERF.B8*	2.22
	Solyc02g022920.1.1	*Ethylene-responsive nuclear protein*	4.1
	**Gibberellin-related DEGs**
	Solyc01g058030.2.1	*Gibberellin 2-oxidase 6*	-6.29
	Solyc01g108570.3.1	*GID1 L2*	-1.45
	Solyc01g079370.3.1	*RGA-like 2*	1.42

Auxin is crucial for triggering ripening and impacts the transition between the two ET production systems (Kumar et al., 2014). DEGs related to auxin transport, signaling, response, and homeostasis responded differentially in the hormone-treated fruits (**Table 2**). In ET-treated fruits, transcripts of *GRETCHEN HAGEN 3 (GH3)*, which converts active auxins to inactive auxin-amino acid conjugates (Staswick et al., 2005), were induced, whereas these genes were downregulated after GA and GA+ET treatments (**Table 2**). Additionally, GA treatment induced *IAA-leucine-resistant-like 2 (ILR2)*, which releases free IAA by cleaving IAA-amino acid conjugates (Bitto et al., 2009; **Table 2**), indicating auxin levels were oppositely regulated by the hormone treatments. Notably, auxin biosynthetic genes *(YUCCAs and tryptophan aminotransferase)* were downregulated in all the treatments, suggesting that auxin biosynthesis may not be affected by the hormone treatments at this stage of ripening (**Table 2**). *Aux/IAA (IAA)* family genes repress the expression of genes in the auxin signaling pathway by interfering with auxin response factor *(ARF)* activity (Wang & Estelle, 2014). GA treatment downregulated the expression of *IAA13* and *IAA16*, whereas ET treatment induced *IAA1*. Moreover, several *small auxins up-regulated RNAs (SAURs)*, which are early auxin-responsive genes that could regulate fruit ripening by integrating auxin signals into ET signals (Pei et al., 2019), were differentially modulated after the hormone treatments (**Table 2**). The effect of hormone treatment on *GH3s, IAA1, IAA16*, and *ARF16* was further confirmed, although the expression levels were not statistically different between the hormone treatment for *GH3-9, IAA1*, and *ARF16*, by quantitative real-time PCR (**Figure S2**).

**Table 2:** Auxin-related differentially expressed genes expressed in tomatoes with hormone treatment.

Gene ID	Annotation	Fold change (log 2 ratio)
**Ethylene**		
Solyc09g091090.2.1	*YUCCA 3*	-2.15
Solyc06g053260.1.1	*SAUR-like auxin-responsive protein family*	-1.53
Solyc03g112460.3.1	*Tryptophan aminotransferase-related protein 2*	-1.2
Solyc01g110560.3.1	*SAUR-like auxin-responsive protein family*	-1.03
Solyc01g091030.3.1	*SAUR-like auxin-responsive protein family*	1.11
Solyc09g083280.3.1	*IAA1*	1.21
Solyc02g064830.3.1	*Auxin-responsive GH3 family protein*	1.24
Solyc03g082510.1.1	*SAUR-like auxin-responsive protein family*	1.25
Solyc01g110843.1.1	*SAUR-like auxin-responsive protein family*	1.42
Solyc04g007690.3.1	*SlPIN3*	1.44
Solyc07g053030.3.1	*GH3-8*	1.47
Solyc10g018340.1.1	*SAUR-like auxin-responsive protein family*	2.26
**Gibberellin**		
Solyc08g079150.1.1	*SAUR-like auxin-responsive protein family*	-2.97
Solyc08g082630.3.1	*Auxin response factor 18*	-2.93
Solyc06g065630.3.1	*Flavin-binding monooxygenase family protein*	-2.16
Solyc07g063850.3.1	*GH3-9*	-1.44
Solyc01g097290.3.1	*IAA16*	-1.2
Solyc09g090910.2.1	*IAA13*	-1.07
Solyc06g072650.1.1	*SAUR-like auxin-responsive protein family*	-1.01
Solyc09g007810.3.1	*Auxin response factor 16*	-1
Solyc05g006220.3.1	*IAA-leucine resistant (ILR)-like 2*	1.17
Solyc08g079140.1.1	*SAUR-like auxin-responsive protein family*	1.48
Solyc11g013310.2.1	*like AUX1 3*	1.49
Solyc06g059730.2.1	*SlPIN6*	3.33
**Gibberellin + Ethylene**		
Solyc01g107400.2.1	*Auxin-responsive GH3 family protein*	-4.66
Solyc01g112100.3.1	*Flavin-dependent monooxygenase 1*	-3.29
Solyc01g110710.3.1	*SAUR-like auxin-responsive protein family*	-3.27
Solyc01g110780.1.1	*SAUR-like auxin-responsive protein family*	-2.6
Solyc00g212260.2.1	*Auxin-responsive GH3 family protein*	-1.09
Solyc01g095580.3.1	*Auxin-responsive GH3 family protein*	-1.09
Solyc02g062230.1.1	*SAUR-like auxin-responsive protein family*	1
Solyc00g188857.1.1	*Auxin response factor 6*	1.19
Solyc01g110920.3.1	*SAUR-like auxin-responsive protein family*	1.46
Solyc01g102540.3.1	*SUPPRESSOR OF AUXIN RESISTANCE1*	1.72
Solyc01g110890.1.1	*SAUR-like auxin-responsive protein family*	2.29
Solyc01g111310.3.1	*like AUXIN RESISTANT 2*	3.05
Solyc01g110770.2.1	*SAUR-like auxin-responsive protein family*	3.42
Solyc01g110860.1.1	*SAUR-like auxin-responsive protein family*	4.29

### Hormone-induced changes in metabolic profiles

**Figure 3:**
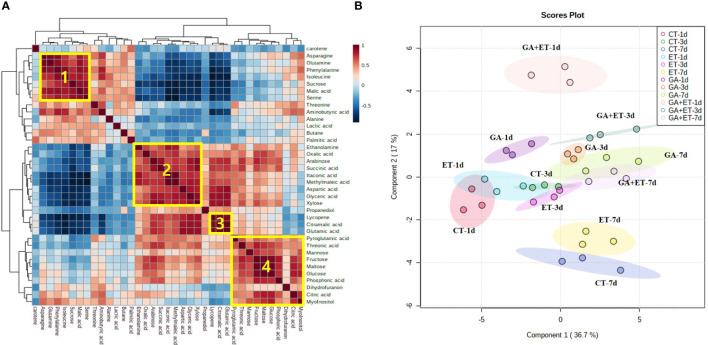
Metabolomic changes in tomatoes with hormone treatment. Combined correlation and cluster analysis **(A)** and Partial least squares discriminant analysis **(B)** of the metabolite profiles of hormone-treated tomatoes. The marked blocks refer to strongly correlated metabolites that show a similar behavior over time. CT, Control; ET, ethylene; GA, gibberellin; GA+ET, the combined hormone treatment.

**Figure 4:**
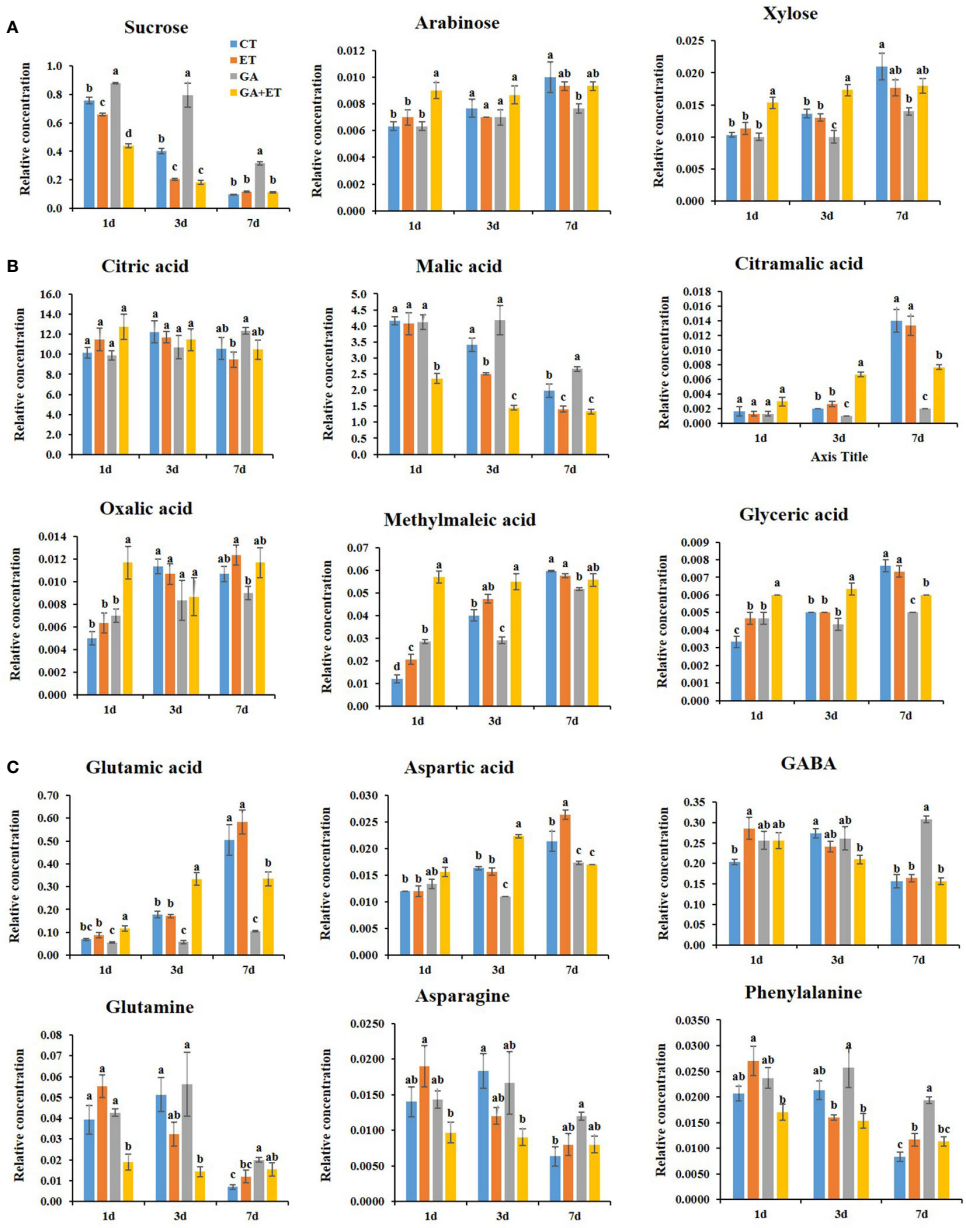
Differential accumulation of ripening-associated metabolites in hormone-treated tomatoes. Sugars **(A)**; Organic acids **(B)**; Amino acids **(C)**. Metabolite contents were identified and quantified by GC-MS. Relative amounts of the metabolites were calculated by using fluoranthene as an internal standard. Error bars represent standard error, and different letters on the graphs represent significant differences between the control and hormone treatments (Student’s *t*-test, *P* < 0.05). CT, Control; ET, ethylene; GA, gibberellin; GA+ET, the combined hormone treatment.

Metabolite profiles were analyzed in pericarp tissues 1, 3, and 7 d after the hormone treatments. The hormone treatments differentially induced the accumulation of different metabolites, such as sugars, organic acids, and amino acids (**Figures 3**, **4**, **Figures S3, S4**). Correlation analysis revealed statistically significant connections between metabolites accumulated during tomato ripening (**Figure 3A**). Four blocks of metabolites have been marked in **Figure 3**. Block 1 indicates a group of metabolites that tended to gradually decrease during ripening. While block 2 metabolites showed gradual increase during ripening, the metabolites from block 3 sharply increased on day 7. Block 4 metabolites were maintained during ripening (**Figure 3A**). The results of PLS-DA distinguished differential metabolite accumulations in tomatoes after the hormone treatments (**Figure 3B**).

Sucrose content declines during fruit ripening, whereas soluble sugar content increases. (Carrari et al., 2006). Tomatoes treated with ET alone or combined with GA displayed lower sucrose content than controls with a sharp decline on until day 3 (**Figure 4A**). However, GA treatment maintained high sucrose levels and delayed the decline in sucrose content for 4d. Consistent with lower sucrose levels, glucose and fructose levels were increased, although not statistically significant, on day 1 in ET- and GA+ET-treated fruits (**Figure S4**). Furthermore, arabinose and xylose were increased in all the treatments during ripening. However, GA treatment reduced the accumulation of these sugars particularly on day 7 (**Figure 4A**). The positive effect of ET and GA+ET or negative effect of GA on the accumulation of mannose, maltose, and myoinositol was largely limited to day 1, respectively (**Figure S4**).

Organic acids accumulate during fruit development and are consumed as respiratory substrates. Malic acid and citric acids are the most abundant organic acids in both climacteric and non-climacteric ripe fruits (Carrari et al., 2006). During the ripening course, ET and GA+ET treated fruits showed lower levels of malic acid than control fruits, whereas GA treatment maintained higher malic acid levels (**Figure 4B**). Malic acid tended to decline on day 3 in control and ET-treated fruits, whereas this decline was delayed for 4 d in GA treated fruits (**Figure 4B**). Additionally, GA-treated fruits displayed higher levels of citric acid and oxalic acid, particularly at 7 d, whereas ET-treated fruits recorded the lowest levels of these metabolites. In the combined treatment, citric acid and oxalic acid levels were similar to controls (**Figure 4B**). Furthermore, GA inhibited the accumulation of citramalic acid, methylmaleic acid, and glyceric acid from day 3–7 (**Figure 4B**). While GA-treated fruits recorded the lowest levels of citramalic acid, methylmaleic acid, and glyceric acid from day 3–7, in ET and GA+ET treated fruits, these organic acid levels were maintained greater than or equal to controls (**Figure 4B**). Similar hormonal effect was observed on the accumulation of itaconic acid, succinic acid, and palmitic acid on day 3 (**Figure S4**). Furthermore, pyroglutamic acid and threonic acid were more responsive to combined hormone treatment than to individual hormone treatments on day 3 (**Figure S4**). No specific trend was observed in the accumulation of phosphoric acid and lactic acid after the hormone treatments (**Figure S4**).

The amino acids glutamic acid and aspartic acid levels are increased during ripening (Kumar et al., 2014). This accumulation in ET and GA+ET treated fruits was greater than or equal to that of controls. However, GA treatment significantly lowered the levels of these amino acids (**Figure 4C**). The gamma-aminobutyric acid (GABA) content was declined by 7 d in all the treatments expect GA. The metabolite accumulations for glutamine, asparagine, phenylalanine, isoleucine, and serine were reduced by 7 d in all the treatments. While ET and GA+ET treatments accelerated the decline in these metabolites, GA treatment inhibited their accumulation (**Figures 4C**, **S4**). Furthermore, threonine levels were specifically increased by GA treatment, whereas alanine accumulation was induced by GA and GA+ET treatments (**Figure S4**).

## Discussion

Fruit ripening is a genetically coordinated process marked by significant biochemical changes in color, texture, flavor, aroma, and nutritional content that coincide with seed maturation. It is precisely regulated by a complex hormonal network. Exogenous hormone treatments have been widely used to study their effects on tomato fruit ripening. Tomato fruits treated with exogenous abscisic acid, jasmonic acid and brassinosteroids displayed early ripening, whereas tomato fruits treated with GA_3_ and auxin showed delayed ripening phenotype (Kumar et al., 2014; Li et al., 2019). Here, we treated tomatoes with these ET and GA and the hormonal effect was determined at transcriptome and metabolite level.

In agreement with previous studies, ET treatment accelerated ripening and GA treatment delayed, as evidenced by the opposite effect of the hormone treatments on fruit quality parameters (color break, a* values, firmness, and lycopene content) (Dostal & Leopold, 1967; Kumar et al., 2014; Li et al., 2017; Li et al., 2019; **Figure 1A-D**). The effect of the combined hormone treatment was in between ET and GA alone treatments (**Figure 1**). Furthermore, ET alone or in combination with GA reduced time to the climacteric rise of ET in concomitant with the upregulation of DEGs related to ET biosynthesis genes (**Figure 1E**; **Table 1**). Delayed fruit reddening after combined hormone treatment, despite endogenous ET levels comparable to ET-treated fruits, may indicate the inhibitory effect of GA over ET on fruit color (**Figures 1A, E**). Furthermore, GA, or in combination with ET, enhanced endogenous GA levels (**Figure 1F**). Cellular GA levels are controlled through the regulation of GA biosynthesis and catabolism. Exogenous GA might have reduced GA biosynthesis, as evidenced by the downregulation of the GA biosynthetic gene *GA20ox* (**Table 1**). ET treatment induced the expression of genes involved in both GA biosynthesis and catabolism. Moreover, DEGs related to GA catabolism were suppressed rather than induced in GA or GA+ET treated fruits, indicating complex regulation of GA homeostasis during tomato ripening (**Table 1**).

### GA alone or in combination with ET can affect ET signaling

*ERFs* are downstream components of ET signaling which regulate the expression of ethylene‐responsive genes. Tomato genome harbors 77 ERFs, of which, 27 exhibit increased expression at the onset of ripening, while 28 display a ripening-associated decrease in expression (Liu et al., 2016). ET treatment positively affected ET signaling, as evidenced by the upregulation of ET biosynthesis and signaling genes (**Table 1**). GA alone or combined with ET may not completely inhibit ET production as *ACS2* and *ACO6* were not repressed in GA- and GA+ET-treated fruits, respectively. However, ET signaling and response were inhibited in these fruits (**Table 1**). For instance, the expression of *ERF.C2*, which downregulated during ripening and negatively correlated with trans-lycopene accumulation in tomatoes (Lee et al., 2012), was repressed after GA treatment (**Table 1**). Similarly, *ERF.C3* that express in mature green tomatoes, was induced by GA treatment (Liu et al., 2016; **Table 1**). Additionally, key ET signaling components, *EIN3* and *EIL* were repressed in the GA+ET-treated fruits. Furthermore, *ERN*, which acts downstream of *EIN3* and negatively regulates ET response (Trentmann, 2000), was strongly induced in the combined hormone-treated fruits (**Table 1**). This scenario suggests that GA alone or combined with ET may negatively affect ET signaling.

### ET and GA treatments oppositely regulate auxin signaling and homeostasis during tomato ripening

Auxin acts as a ripening inhibitor, and low levels of auxin are essential for triggering ripening in tomatoes (Buta & Spaulding, 1994). Several auxin conjugation or degradation mechanisms can be used to reduce auxin levels (Woodward & Bartel, 2005). In the GA-treated fruits, the expression of *GH3s*, which converts active auxins to inactive auxin-amino acid conjugates (Staswick et al., 2005), was repressed, whereas that of *ILR2*, which releases free IAA by cleaving IAA-amino acid conjugates (Bitto et al., 2009), was induced (**Table 2**). In contrast, *GH3* expression was induced by ET treatment (**Table 2**). GA treatment might have helped maintain high levels of auxin by regulating amino acid–auxin conjugation and auxin transport. In the combined treatment groups, the expression of *GH3* was downregulated, whereas that of the auxin importer like aux 2 (Péret et al., 2012) was upregulated (**Table 2**), highlighting the tight regulation of auxin homeostasis in the pericarp cells of tomatoes treated with GA+ET. The role of *GH3s* in fruit ripening has been reported in different fruits (Kumar et al., 2014). Notably, auxin biosynthetic genes (*YUCCAs* and *tryptophan aminotransferase*) were downregulated in all the treatments, suggesting that auxin biosynthesis is not part of the regulation of fruit ripening (**Table 2**). IAA family genes repress the expression of genes in the auxin signaling pathway by interfering with *ARF* activity (Wang and Estelle, 2014). In the GA-treated fruits, *IAA13* and *IAA16* expressions were downregulated. In contrast, *IAA1* expression was induced in the ET-treated fruits (**Table 2**). Thus, the high auxin levels in the GA-treated fruits might have induced auxin signaling, indicating mediation of auxin signaling in GA-induced delayed fruit ripening. Additionally, *ARF6*, which is involved in auxin and GA interaction (Liu et al., 2018), was upregulated in the combined hormone-treated fruits (**Table 2**). Furthermore, several *SAURs*, which are early auxin-responsive genes that could regulate fruit ripening by integrating auxin signals into ET signals (Pei et al., 2019), were differentially modulated after the hormone treatments (**Table 2**), indicating the potential involvement of auxin signaling in ET and GA interactions during fruit ripening. The effect of hormone treatment on *GH3s*, *IAA1*, *IAA16* and *ARF16* was confirmed, although the expression levels were not statistically different between the hormone treatment for *IAA1* and *ARF16* by quantitative real-time PCR (**Figure S2**).

### GA treatment affects the accumulation of ripening-associated metabolites

The ripening process involves profound changes in key metabolites. Sugars accumulate mainly due to sugar import or from starch degradation during ripening. Furthermore, sucrose can be metabolized into glucose and fructose (Carrari et al., 2006). In this study, ET alone or in combination with GA reduced sucrose levels during ripening, whereas this reduction was prevented in GA-treated fruits (**Figure 4A**). Additionally, GA-treated fruits showed lower levels of arabinose and xylose, the cell wall sugars that tend to accumulate during ripening (Takizawa et al., 2014), than that of control and ET-treated fruits (**Figure 4A**). Although the GA effect was restricted largely to 1 d, accumulation of glucose, fructose, and several other sugars were inhibited by GA treatment (**Figure S4**). This suggests ripening-associated changes in sugar metabolism are delayed in GA-treated fruits. Moreover, sugar accumulation in the combined treatment groups was greater than or equal to that of ET-treated fruits (**Figure 4A**, **Figure S4**) may suggest the dominance of ET positive effect over GA inhibitory effect on sugar metabolism during ripening.

Organic acids make an important contribution to the taste and overall quality of tomatoes. Citric acid and malic acid are major fruit acids that decrease during ripening progression (Carrari et al., 2006). In this study, the decline in citric acid and malic acid levels was accelerated in ET-treated fruits (**Figure 4B**). The metabolism of citric acid and malic acid was demonstrated to be regulated under ET regulation. The content of these acids was higher in transgenic antisense *LeACS2* lines and returned to normal levels after transgenic fruits were treated with ET (Gao et al., 2007). However, the decline in citric and malic acids was inhibited by GA treatment (**Figure 4B**), indicating an altered metabolic shift in GA-treated fruits. Notably, GA+ET treatment did not affect citric acid accumulation but significantly affected malic acid decline (**Figure 4B**). López et al. (2015) reported that manipulation of malic acid concentration in tomato fruits altered their postharvest behavior at room temperature. Moreover, an association between malate content, fruit firmness, and the shelf-life of tomatoes has been suggested (López et al., 2015). The presence of high malic acid levels and lower accumulation of cell wall sugars arabinose and xylose may partly explain firmness control in GA-treated fruits. Additionally, GA treatment inhibited ripening-associated increases in citramalic acid, oxalic acid, methyl maleic acid, and glyceric acids (**Figure 4B**). Citramalic acid, which can be produced from pyruvate and acetyl-CoA through a series of recursive reactions, is one of the indicators of metabolic shift during ripening (Oms-Oliu et al., 2011), which peaked on 7d in control and ET-treated fruits (**Figure 4B**). However, this metabolite content was reduced in GA- and GA+ET-treated fruits (**Figure 4B**). Furthermore, oxalic acid accumulation, which weakens the cell wall due to chelation of Ca2+ (Bateman & Beer, 1965), was inhibited in the GA-treated fruits (**Figure 4B**). These results indicate altered organic acid metabolism in the GA-treated fruits.

In tomatoes, free amino acids increase dramatically during fruit ripening, and their abundance changes differentially (Carrari et al., 2006). Glutamic acid, aspartic acid, and GABA are the most quantitatively important amino acids. As the fruit ripens, levels of GABA decline, and glutamic acid and aspartic acid content accumulate (Carrari et al., 2006). In this study, GABA content declined at 7d in all the treatments except in GA-treated fruits (**Figure 4C**). Furthermore, glutamic acid and aspartic acid levels peaked at 7 d. However, GA and GA+ET treatments tremendously inhibited the accumulation of these metabolites (**Figure 4C**). As glutamic acid is a direct precursor of chlorophyll, its accumulation in ET-treated fruit may be linked to downregulation of chlorophyll synthesis (Carrari et al., 2006). Additionally, GA treatment inhibited decline in other amino acids, such as glutamine, asparagine, phenylalanine, isoleucine, and serine (**Figure S4**). This implies that GA affects the accumulation of key ripening-associated amino acids.

PLS-DA showed that the overall metabolic profiles of the combined hormone-treated fruits were distinctly separated from those of the individual treatments on days 1 and 3 but overlapped on day 7 with the day-3 profiles of ET- and GA-treated fruits (**Figure 3B**). Additionally, the overall metabolic profiles of the combined treatment appeared to be closer to those of GA-treated fruits (**Figure 3B**). Similarly, Tobaruela et al. (2021) reported that the overall profiles of the volatile organic compounds of Micro-Tom tomatoes treated with a combination of ET and auxin were closer to the auxin profiles at full ripening stage. The analysis of changes in individual metabolites showed that the inhibitory effect of GA treatment may be repressed when GA is combined with ET. For instance, the levels of citric acid, glyceric acid, and glutamic acid in GA+ET-treated fruits intermediated between those of GA- and ET-treated fruits on day 7 (**Figures 4B, C**). Furthermore, ET may completely diminish the repressor effect of GA on specific metabolites. For instance, levels of sucrose, arabinose, xylose, malic acid, GABA, and palmitic acid on day 7 in GA+ET-treated fruits were similar to those of ET-treated fruits (**Figures 4**, **S4**). On the other hand, the addition of ET to GA treatment may not affect GA inhibitory effect on some metabolites, as evidenced by the similar aspartic acid levels in GA- and GA+ET-treated fruits on day 7. Interestingly, adding ET to GA treatment may have an additive effect on the activity of ET. For instance, the decline in sucrose and malic acid levels on days 1 and 3, respectively, in GA+ET-treated fruits was greater than in ET-treated fruits. Taken together, further studies are needed to understand the complex interaction between ET and GA during fruit ripening.

## Conclusion

**Figure 5:**
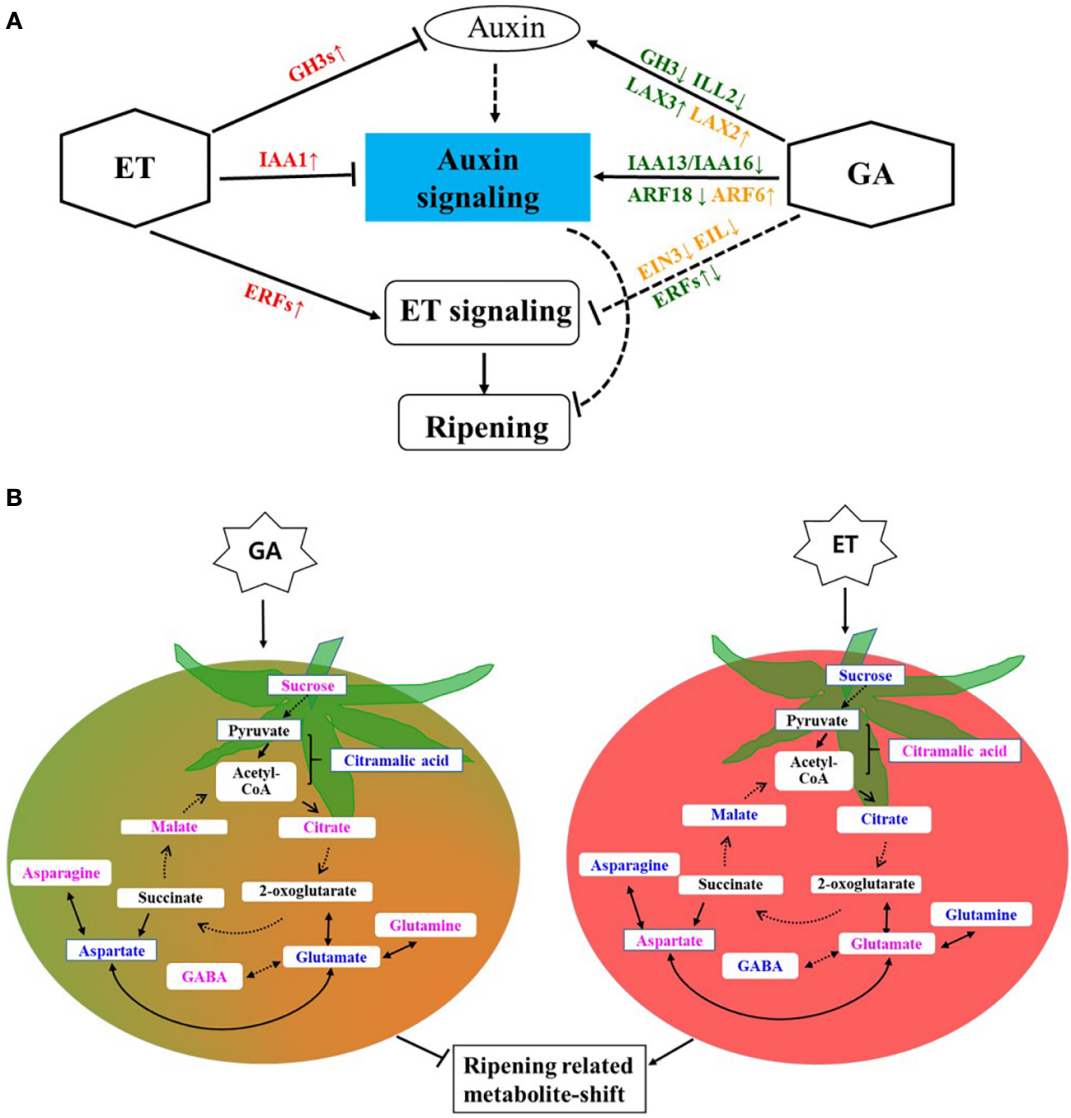
Presumptive model of ET–GA interaction and its potential effect on metabolic shift during tomato ripening. **(A)** ET–GA interaction at transcriptomic level. Names of genes in red, green, and orange colors indicate their upregulation (upward arrow) or downregulation (downward arrow) in ET, GA, or their combination, respectively. Solid and blunt arrows represent positive and negative regulation, respectively. Blunt dashed arrows indicate indirect negative regulation. ET, Ethylene and GA, gibberellin. **(B)** ET–GA interaction at metabolite level. Names of metabolite in blue and pink colors indicate their decreased or increased accumulation after ET and GA treatments, respectively. Round Dot arrows indicate multiple steps. Ethylene, ET and gibberellin, GA.

GA and ET may critically interact with auxin during ripening (**Figure 5A**). GA treatment may regulate cellular auxin levels by controlling *GH3*-mediated auxin conjugation, resulting in the induction of auxin signaling, which involves the suppression of *IAAs*. Auxin signaling and response involving *ARFs* and *SAURs* may potentially suppress ET response. At the onset of ripening, the climacteric rise of ET may induce the expression of *GH3s*, resulting in low active cellular auxin and leading to the suppression of auxin signaling and induced ET response. At the metabolite level, GA-induced auxin signaling may translate to the inhibition of ripening-associated metabolite shift (**Figure 5B**). ET treatment induced known metabolite changes, such as those in sugar levels and accumulation of amino acids glutamate and aspartate and storage associated organic acids, such as citric acid, malic acid citramalic acid. In contrast, GA treatment helped maintain high levels of sucrose and GABA, thereby delaying ripening and senescence. In conclusion, GA partially regulates metabolic changes during ripening by controlling auxin conjugation and signaling. Our findings will be useful for further understanding interaction between ET and GA during fruit ripening.

## Data availability statement

The datasets presented in this study can be found in online repositories. The names of the repository/repositories and accession number(s) can be found here: https://www.ncbi.nlm.nih.gov/Accession: PRJNA893839.

## Author contributions

Conceptualization, SM and M-HP. Writing¬–original draft preparation, SM and M-HP. Supervision, M-HP. All authors contributed to the article and approved the submitted version.

## Funding

This study was funded by the Cooperative Research Program for Agriculture, Science, and Technology (Project No. PJ01502903) in the Rural Development Administration of the Republic of Korea.

## Conflict of interest

The authors declare that the research was conducted in the absence of any commercial or financial relationships that could be construed as a potential conflict of interest.

## Publisher’s note

All claims expressed in this article are solely those of the authors and do not necessarily represent those of their affiliated organizations, or those of the publisher, the editors and the reviewers. Any product that may be evaluated in this article, or claim that may be made by its manufacturer, is not guaranteed or endorsed by the publisher.
