# Supplementary material for: Gibberellin delays metabolic shift during tomato ripening by inducing auxin signaling
Source: Front Plant Sci. 2022 Nov 14;13:1045761. doi: 10.3389/fpls.2022.1045761 (PMC9703062; doi:10.3389/fpls.2022.1045761)
Supplement: Supplementary file 1 [file DataSheet_1.docx]

Supplementary Material

## Supplementary Tables

**Table S1** Primer sequences for quantitative RT-PCR amplification.

| Gene | Accession | Forward | Reverse |
| --- | --- | --- | --- |
| *GH3-8* | Solyc07g053030.3 | TCGTTCATCCATCCTTTGTTCT | ACCTTCTCCCTAGCTCTTCTT |
| *GH3-9* | Solyc07g063850.3 | ACGAACTACACAAGCCCTAAC | CAGCACCCACACGAAGAA |
| *ARF16* | Solyc09g007810.3 | CCTTGTAAAGTCTCCGCGATTA | TCAACCTCATTCCTACCAACAG |
| *IAA1* | Solyc09g083280.3 | ATGACAAGTATAGTGGGCAATCA | TCTGTACCAGGCAAACCTAATC |
| *IAA16* | Solyc01g097290 | TTGACGGTGCACCATACTTAC | TCCTTGAGTCCCACAATTTCC |
| *Actin* | AB199316 | AAGGATGCGTATGTGGGTGA | TTAAGGGGTGCCTCAGTCAG |

##
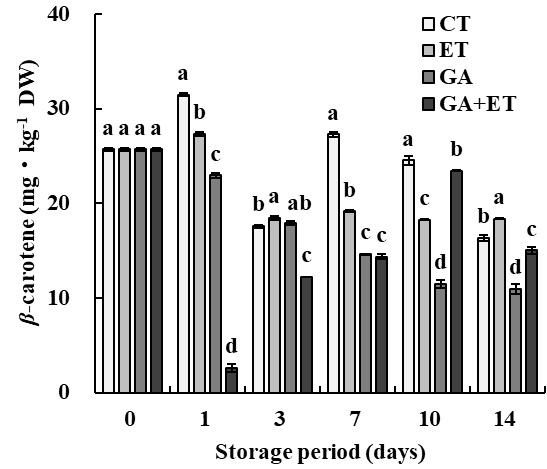
Supplementary Figures

**Figure S1.** Effect of hormone treatments on β-carotene content in tomato. Error bars represent standard error, and different letters on the graphs represent significant differences between the control and hormone treatments (Student’s t-test, P < 0.05). CT, Control; ET, ethylene; GA, gibberellin; GA+ET, the combined hormone treatment.

**
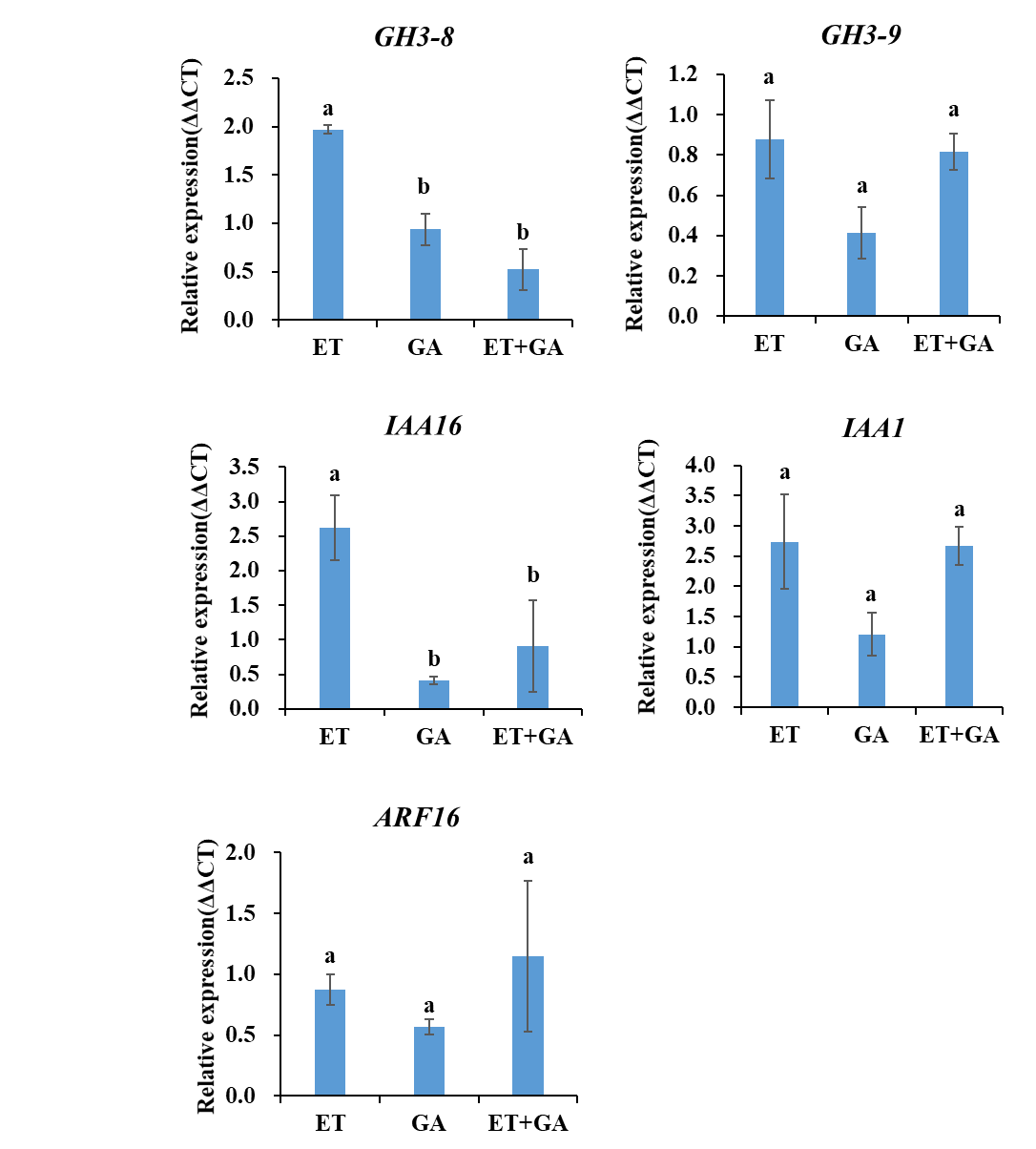
**

**Figure S2**. Validation of auxin-related differentially expressed genes through quantitative real-time PCR. Error bars represent standard error, and different letters on the graphs represent significant differences between the control and hormone treatments (Student’s t-test, P < 0.05). ET, Ethylene; GA, gibberellin; GA+ET, the combined hormone treatment.

**
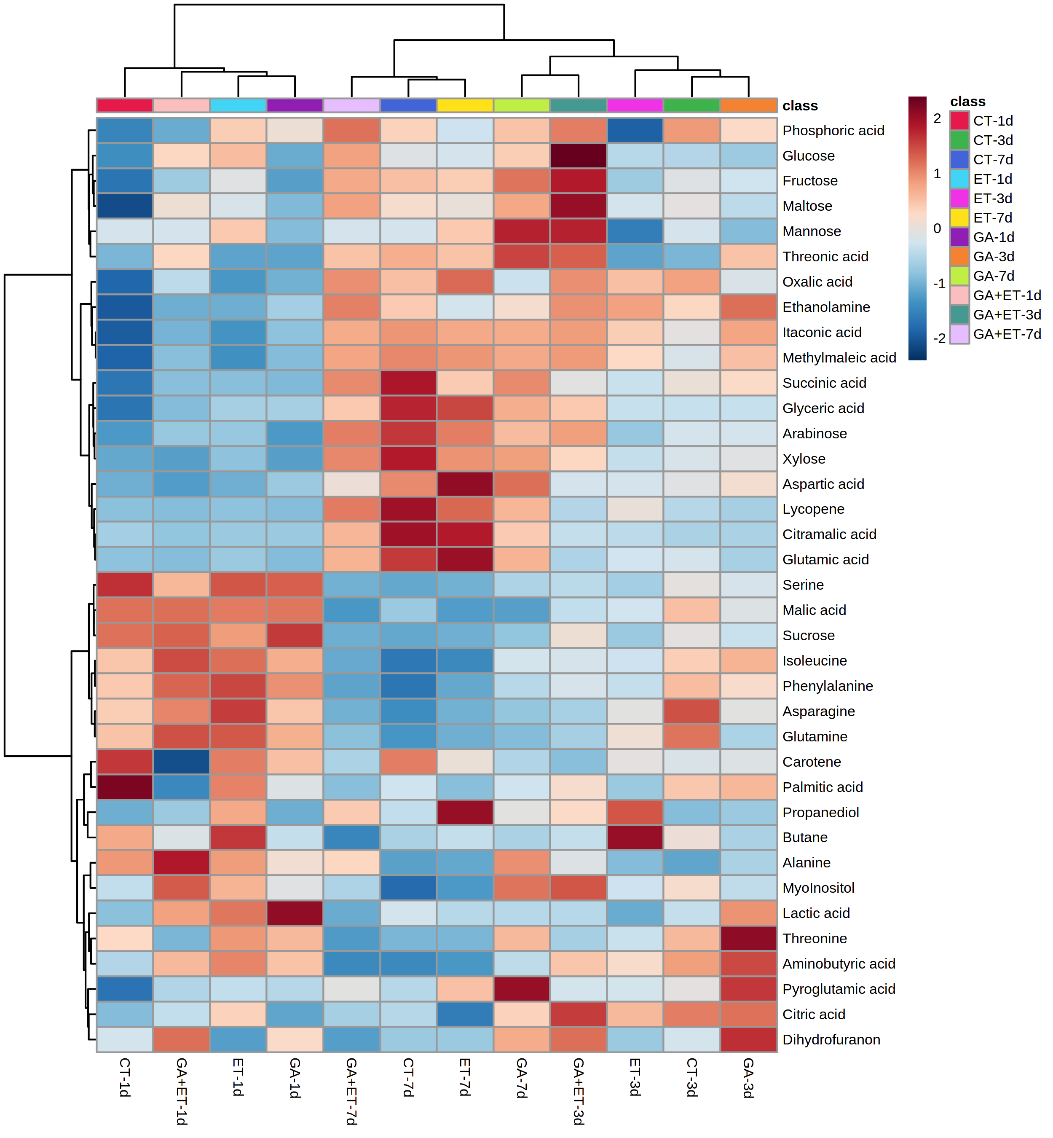
Figure S3.** Heat map of metabolomics data from tomatoes with hormone treatment. Metabolite contents were identified and quantified by GC-MS. CT, Control; ET, ethylene; GA, gibberellin; GA+ET, the combined hormone treatment.

**
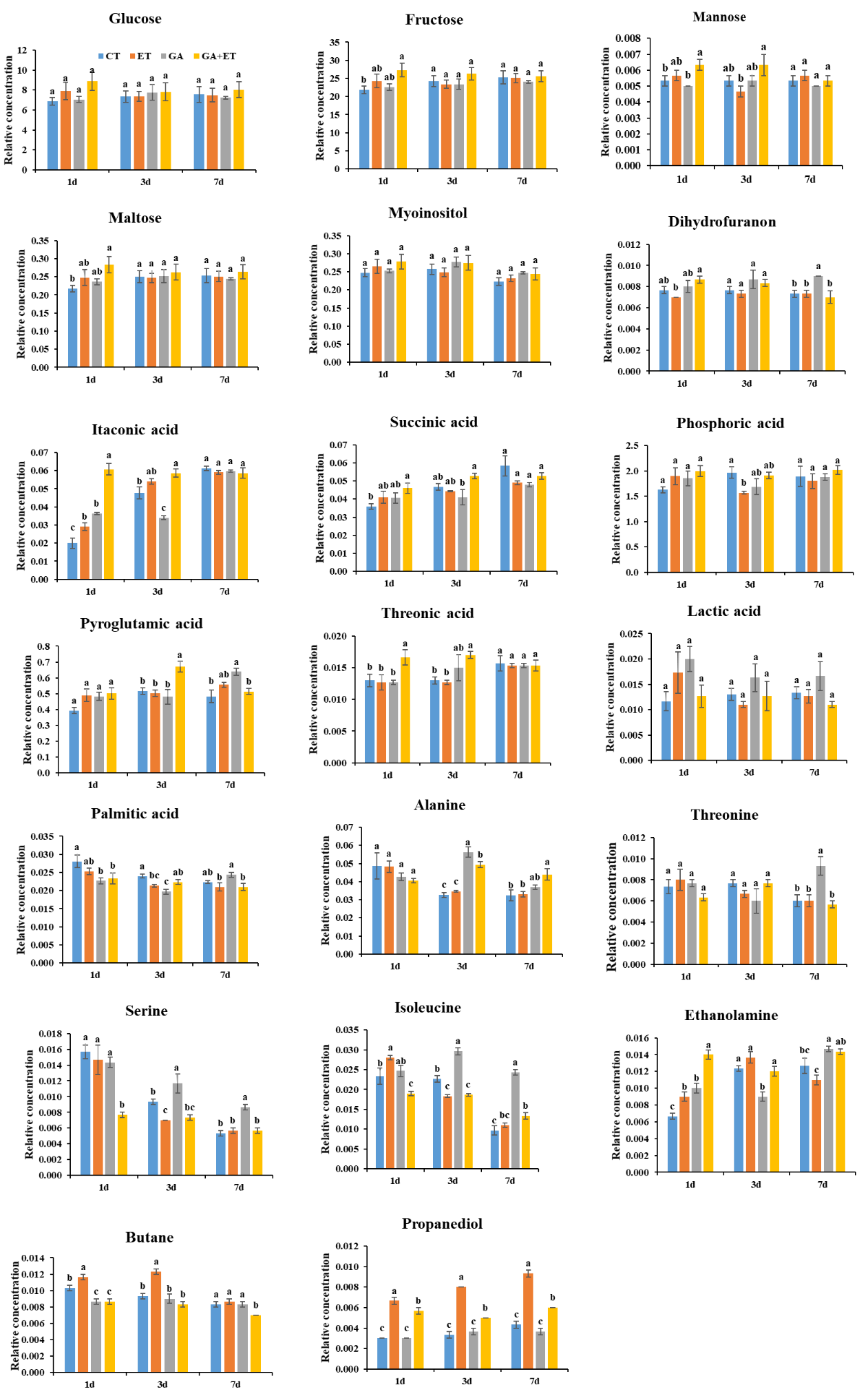
**

**Figure S4.** Differentially accumulated metabolites in tomatoes with hormone treatment. Metabolite contents were identified and quantified by GC-MS. The relative concentration on the y-axis shows the normalized value based on fluoranthene as an internal standard. Error bars represent standard error, and different letters on the graphs represent significant differences between the control and hormone treatments (Student’s *t*-test, P < 0.05). CT, Control; ET, ethylene; GA, gibberellin; GA+ET, the combined hormone treatment.
